# EdgeVidCap: A Channel-Spatial Dual-Branch Lightweight Video Captioning Model for IoT Edge Cameras

**DOI:** 10.3390/s25164897

**Published:** 2025-08-08

**Authors:** Lan Guo, Xuyang Li, Jinqiang Wang, Jie Xiao, Yufeng Hou, Peng Zhi, Binbin Yong, Linghuey Li, Qingguo Zhou, Kuanching Li

**Affiliations:** 1 School of Information Science and Engineering, Lanzhou University, Lanzhou 730000, China; guol2023@lzu.edu.cn (L.G.); lxuyang2023@lzu.edu.cn (X.L.); jqwang16@lzu.edu.cn (J.W.); xiaoj2024@lzu.edu.cn (J.X.); 120220909101@lzu.edu.cn (Y.H.); zhip21@lzu.edu.cn (P.Z.); yongbb@lzu.edu.cn (B.Y.); zhouqg@lzu.edu.cn (Q.Z.); 2 Department of Computer Science and Information Engineering, Providence University, Taichung 43301, Taiwan; s1091858@gm.pu.edu.tw

**Keywords:** edge computing, video captioning, IOT, lightweight neural networks, attention mechanism, state space models

## Abstract

With the deep integration of edge computing and Internet of Things (IoT) technologies, the computational capabilities of intelligent edge cameras continue to advance, providing new opportunities for the local deployment of video understanding algorithms. However, existing video captioning models suffer from high computational complexity and large parameter counts, making them challenging to meet the real-time processing requirements of resource-constrained IoT edge devices. In this work, we propose EdgeVidCap, a lightweight video captioning model specifically designed for IoT edge cameras. Specifically, we design a hybrid module termed Synergetic Attention State Mamba (SASM) that incorporates channel attention mechanisms to enhance feature selection capabilities and leverages State Space Models (SSMs) to efficiently capture long-range spatial dependencies, achieving efficient spatiotemporal modeling of multimodal video features. In the caption generation stage, we propose an adaptive attention-guided LSTM decoder that can dynamically adjust feature weights according to video content and auto-regressively generate semantically rich and accurate textual descriptions. Comprehensive evaluations of EdgeVidCap on mainstream datasets, including MSR-VTT and MSVD are analyzed. Experimental results demonstrate that our system demonstrated enhanced precision relative to existing investigations, and our streamlined frame filtering mechanism yielded greater processing efficiency while creating more dependable descriptions following frame selection.

## 1. Introduction

With the flourishing development of short video platforms and the explosive growth of multimedia content, automatic video captioning technology has demonstrated broad application prospects in domains such as content retrieval, accessibility, and intelligent media processing [1,2]. Video captioning aims to automatically transform video content into accurate and fluent natural language descriptions. This task requires simultaneous understanding of visual content, temporal relationships, and semantic information, making it a significant research topic at the intersection of computer vision and natural language processing [3]. In recent years, with the rapid advancement of edge computing technology and significant improvements in deep neural network performance, intelligent edge devices have been widely deployed in video processing. These intelligent cameras equipped with edge computing capabilities (hereinafter referred to as edge cameras) possess more powerful local computational resources and can achieve real-time video analysis and processing, providing new technological pathways for the practical application of video captioning technology [4,5].

Video captioning technology has evolved from template-based approaches and statistical learning methods to deep learning paradigms. Early studies primarily relied on predefined templates and rules, where objects, actions, and scenes detected in videos were used to populate language templates [6]. Subsequently, statistical machine learning methods introduced more flexible language models, but these approaches struggled to handle complex visual-language associations [7]. In the deep learning era, the research paradigm for video captioning has undergone a fundamental transformation. The CNN-RNN architecture pioneered end-to-end caption generation [8], where Convolutional Neural Networks (CNNs) extract visual features and Recurrent Neural Networks (RNNs) perform sequence decoding. However, such methods often overlook the long-term temporal dependencies within videos. To better model temporal information, researchers have proposed various improvements: the introduction of attention mechanisms enables models to dynamically focus on essential parts of a video [9]; temporal attention networks further enhance the modeling of action and event evolution [10,11]; and hierarchical attention structures facilitate multi-scale feature extraction from frame-level to segment-level [12,13]. With the breakthrough progress of Transformers across various domains [14,15], video captioning methods based on self-attention mechanisms have gradually emerged as a research hotspot. Although these approaches demonstrate superior performance in modeling long-range dependencies, Vision Transformers (ViTs) are constrained by the quadratic computational complexity of their self-attention mechanisms, rendering them difficult to deploy in real-world scenarios with limited computational resources. Existing methodologies still suffer from the following critical issues: (1) redundant video information, high model computational complexity, and lack of targeted keyframe selection strategies; (2) insufficient multimodal feature fusion, suboptimal intermodal alignment, and excessive model parameters.

To address these aforementioned challenges, we propose EdgeVidCap, a channel-spatial dual-branch lightweight video captioning model for IoT edge cameras, incorporating cross-attention mechanisms into the keyframe selection process, enabling efficient extraction of salient information from video sequences. In terms of feature representation, the model integrates miniGPT-based visual object features, deep audio features, and 3D optical flow motion features to construct comprehensive multimodal representations. During the feature fusion stage, a hybrid module termed Synergetic Attention State Mamba (SASM) is designed, which introduces channel attention mechanisms to enhance feature selection capabilities while incorporating State Space Models (SSMs) to efficiently capture long-range spatial dependencies, thereby achieving efficient spatiotemporal modeling of multimodal video features. In the caption generation phase, an adaptive attention-guided sentence decoder is proposed, which dynamically adjusts feature weights according to contextual information to generate more accurate and natural descriptive text.

Our contributions are as follows.

- To propose a keyframe guidance mechanism that achieves precise localization and extraction of critical information in video sequences through cross-attention computation.
- To design a hybrid module called Synergetic Attention State Mamba (SASM), which incorporates channel attention mechanisms to enhance feature selection capabilities while leveraging State Space Models (SSMs) to efficiently capture long-range spatial dependencies, enabling efficient spatiotemporal modeling of multimodal video features.
- Introduce adaptive attention mechanisms to construct a sentence decoder that achieves selective focus on fused features through dynamic adjustment of feature weights at different temporal instances.

## 2. Related Work

The research on video captioning began with the encoder–decoder framework. Venugopalan et al. [6] first proposed an end-to-end sequence-to-sequence learning model (S2VT), which utilized CNNs to extract video frame features and employed LSTMs for sequence decoding. Building on this, Yao et al. [16] introduced 3D convolutional networks to enhance the representation of spatiotemporal features. Pan et al. [17] designed a hierarchical LSTM network to separately model the temporal structure of videos and the language generation process. Although these methods established the basic paradigm for video captioning, they often faced challenges such as difficulties in modeling long-term dependencies and insufficient utilization of features.

To address these limitations, Krishna et al. [18] proposed a dense video captioning framework that improved the description of long videos through segment-wise processing. Wang et al. [19] designed a bidirectional LSTM structure to better leverage contextual information for guiding caption generation. The introduction of attention mechanisms significantly boosted the performance of video captioning. Early works primarily focused on temporal attention, such as the hierarchical temporal attention network proposed by Yu et al. [20], which adaptively focused on essential time segments in videos. With further advancements, spatial attention also gained attention. Zhang et al. [21] developed a spatiotemporal dual-attention mechanism to achieve fine-grained visual feature extraction.

With the successful application of the Transformer architecture in computer vision, recent research has increasingly focused on transferring the capabilities of large language models to video captioning tasks. Dai et al. [22] proposed a temporal-aware Transformer structure based on visual encoders, which improved the modeling of video dynamics while maintaining efficient inference. Guo et al. [23] proposed a semantic guidance network for video captioning, which effectively improved description accuracy and generalization ability by implementing a novel scene frame sampling strategy to address visual information redundancy and scene information omission. Yang et al. [24] introduced the VideoChat model, which significantly enhanced the naturalness and accuracy of captions by fine-tuning ChatGPT with video understanding instructions. Wang et al. [25] designed the Video-LLaMA framework, injecting video understanding capabilities into the LLaMA model, thereby enabling more flexible video–text interactions.

## 3. Problem Definition

### 3.1. Task Overview

Video captioning is a challenging multimodal task that aims to generate natural language descriptions for video content automatically. Given an input video sequence, the goal is to produce a coherent and semantically accurate textual description that captures the key visual events, objects, and their temporal relationships. This task is particularly challenging for IoT edge cameras due to computational constraints and the need for real-time processing.

### 3.2. System Pipeline Overview

The core objective of video captioning is to summarize and reinterpret video content using natural language. Due to the significant heterogeneity between video and natural language, the data processing involved in this task is highly complex. As such, video captioning represents a major challenge in computer vision. Having established the overall workflow, we now formally define the mathematical representations: Let the training dataset be represented as Data={(Vi,Ti);1≤i≤N}, where $V_{i}$ denotes the i-th video and $T_{i}$ represents its corresponding ground-truth caption. Each video $V_{i}$ is decomposed into a sequence of frames: Frame={fi;1≤i≤S}, where *S* represents the total number of frames. According to the approach in [26], the dataset is divided into training, testing, and validation subsets in 65%, 30%, and 5%, respectively. These subsets are constructed as follows: The training set is as follows: $Data\_train=\{\left(V_{i},T_{i}\right);0<i\leq \left\lfloor 0.65\times N\right\rfloor \}$; the testing set is as follows: $Data\_test=\{\left(V_{i},T_{i}\right);\left\lfloor 0.65\times N\right\rfloor +1\leq i\leq \left\lfloor 0.95\times N\right\rfloor \}$; and the validation set is as follows: $Data\_val=\{\left(V_{i},T_{i}\right);\left\lfloor 0.95\times N\right\rfloor +1\leq i\leq N\}$.

During the data preprocessing stage, each video is first decomposed into *S* frames using the FFmpeg tool, *i.e.*, $F_{i}=\left\{f_{1},f_{2},\ldots ,f_{s}\right\}$. Subsequently, the audio information of each video is extracted to construct an audio set $A=\{a_{1},a_{2},\ldots ,a_{n}\}$. Finally, cross-attention computation is used to obtain *X* keyframes as the input to the model, denoted as follows: $F=\{f_{1},f_{2},\ldots ,f_{x}\}$. During the encoding stage, the sampled frame set *F* is input into 2D-CNN and 3D-CNN models. The 2D-CNN model outputs a *P*-dimensional feature vector, defined as $U_{d}=\left\{ud_{1},ud_{2},\ldots ,ud_{p}\right\}$, while the 3D-CNN model outputs a *Q*-dimensional feature vector, $Z_{d}=\left\{zd_{1},zd_{2},\ldots ,zd_{q}\right\}$. The corresponding audio information extracted from video *A* is input into Google’s pre-trained audio model VGGish [27], which extracts an *R*-dimensional audio feature vector, $W_{d}=\left\{wd_{1},wd_{2},\ldots ,wd_{r}\right\}$. The final textual description generated by the model is represented as follows: $Y=\{y_{1},y_{2},\ldots ,y_{m}\}$.

## 4. Model Construction

### 4.1. Model Architecture

**Figure 1:**
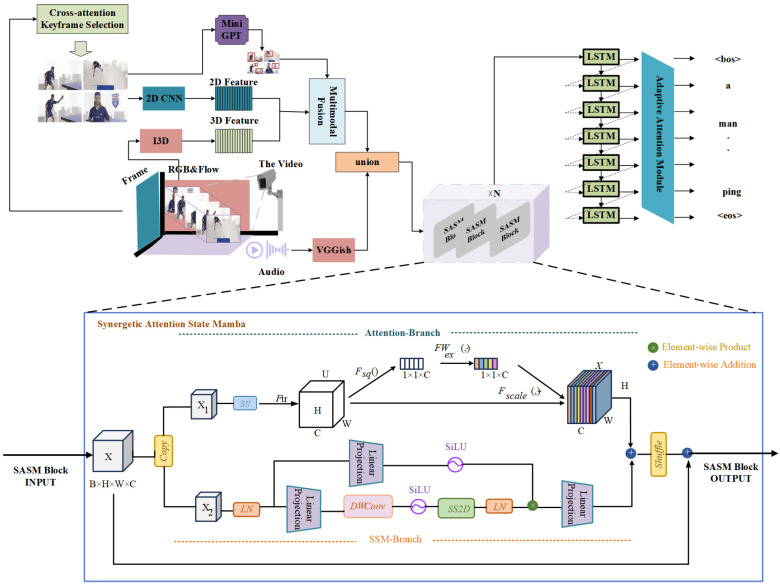
Channel-spatial dual-branch lightweight video description model for IoT edge cameras. Overview of the proposed algorithmic model for video description generation. The input video is decomposed into frames and audio using FFmpeg. Keyframes are selected via a visual span-based mechanism (Section 4.2) to reduce redundancy. Features are extracted using 2D CNN with pruned miniGPT for static visuals, I3D for spatiotemporal visuals, and VGGish for audio. These are fused in a multimodal network (Section 4.3), enhanced by the Synergetic Attention State Mamba Block (Section 4.4) for synergistic optimization, and decoded adaptively (Section 4.5) to produce textual descriptions.

The architecture of our proposed algorithmic model is illustrated in Figure 1. The model takes a video as input, which is first decomposed into a sequence of image frames and a single-channel audio stream using FFmpeg. Given the high similarity between adjacent video frames, directly processing the complete frame sequence incurs substantial redundant computations, resulting in prolonged processing times and the potential introduction of extraneous information. To address this, we introduce a visual span-based keyframe selection mechanism (Section 4.2), which retains only the essential keyframes to supply visual features to the model, thereby effectively reducing computational overhead. In the feature extraction stage, we employ multiple methods to derive features from different modalities: static visual features of keyframes are extracted using a 2D CNN and a pruned miniGPT architecture; spatiotemporal 3D visual features of the video are captured via the I3D network [12]; and deep audio features are obtained from the video’s audio component based on Google’s pre-trained VGGish model [27]. Subsequently, we construct a multimodal fusion network (Section 4.3) to integrate the 2D and 3D visual features, while concatenating the audio features extracted by VGGish. Considering the distinct representational characteristics of visual and audio temporal features, we design the Synergetic Attention State Mamba Block (Section 4.4). This module adopts a dual-branch parallel architecture to achieve synergistic optimization between channel attention mechanisms and spatial state modeling. Finally, the fused encoding vector is fed into the adaptive sentence decoding module (Section 4.5). Within this module, the adaptive attention mechanism processes the hidden states output by the LSTM at each time step, dynamically adjusting the weights assigned to encoding vectors at different moments to enhance focus on critical content across multimodal features. A word probability distribution is then generated via the Softmax function, ultimately yielding a textual description corresponding to the input video.

### 4.2. Visual Span Video Keyframe Selection

**Figure 2:**
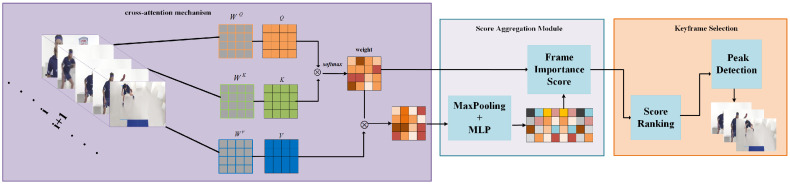
Visual-spanning keyframe selection. The framework processes input video sequences through attention-based mechanisms, computing attention weights between consecutive frames to precisely capture scene transitions. The architecture employs a sparse aggregation module to analyze temporal relationships: when significant differences exist between consecutive frames, the attention distribution exhibits greater dispersion, indicating potential scene transitions; conversely, when frames demonstrate high similarity, attention becomes more concentrated. Through similarity assessment, similar frames can be removed while retaining keyframes with greater differences.

In video content description generation tasks, processing all video frame sequences generates substantial computational redundancy, which is time-consuming and prone to producing partially extraneous information [28,29]. To mitigate these issues, we propose a novel video span keyframe selection methodology, as illustrated in Figure 2. Through the computation of attention weights between consecutive frames, scene transitions can be precisely captured [30]. When significant differences exist between two frames, the attention distribution exhibits greater dispersion; conversely, when two frames demonstrate high similarity, the attention becomes more concentrated. This mechanism facilitates the efficient identification and extraction of semantically significant frames within extended video sequences, thereby reducing computational overhead while preserving critical visual information.

The pseudocode of the algorithm is presented in Algorithm 1. Utilizing the cross-attention mechanism, the algorithm processes a given video comprising *N* sampled frames, denoted as $\left\{f_{1},f_{2},\ldots ,f_{N}\right\}$.
**Algorithm 1** Cross-attention keyframe selection**Input:**  1:*N* video frames $\left\{I_{1},I_{2},\ldots ,I_{N}\right\}$  2:Pre-trained visual model Enc(·)  3:Projection matrices $W^{Q},W^{K},W^{V}$  4:Threshold τ or desired number of keyframes *M***Output:** Keyframe set *K***Steps:**  1:For i=1 to *N* do  2:$F_{i}\leftarrow \mathrm{Enc}\left(l_{i}\right)$  3:End for  4:For i=1 to N−1 do  5:$Q\leftarrow F_{i}W^{Q}$; $K\leftarrow F_{i+1}W^{K}$; $V\leftarrow F_{i+1}W^{V}$  6:$A_{i}\leftarrow \mathrm{softmax}\left(\frac{QK^{T}}{\sqrt{d}}\right)$  7:$s_{i}\leftarrow \mathrm{Pool}\left(A_{i}\right)$  8:$diff_{i}\leftarrow 1-s_{i}$  9:end for10:$K\leftarrow \mathrm{Select}\;\mathrm{frames}\;\left\{l_{i+1}\right\}\;\mathrm{with}\;diff_{i}>\tau \;\mathrm{or}\;\mathrm{top}\;M\;diff_{i}$11:**Return** *K*

We first utilize a visual encoder to extract features from each frame, resulting in frame-level features $F_{i}\in R^{L\times d}$, where *L* denotes the number of patches/tokens into which a frame is divided, and *d* represents the dimensionality of each token’s feature vector. To evaluate scene changes, the differences between consecutive frames $I_{i}$ and $I_{i+1}$ are iteratively assessed.

In the Cross-Attention mechanism, to compute the attention distribution between frames $I_{i}$ and $I_{i+1}$, we treat $I_{i}$ as the Query (*Q*), and $I_{i+1}$ as the Key (*K*) and Value (*V*). By applying learnable linear projection matrices $W^{Q}$, $W^{K}$, and $W^{V}$, the original features are mapped into the attention subspace, as formulated in Equation (1):

(1) $$Q=F_{i}W^{Q},\;K=F_{i+1}W^{K},\;V=F_{i+1}W^{V}$$

The dimensions of Q,K,V are all $R^{L\times d}$, matching the dimensions of the original features while incorporating a new learnable parameter space. Given *Q* and *K*, the attention weight matrix $A_{i}$ can be computed as in Equation (2).

(2) $$A_{i}=\mathrm{softmax}\left(\frac{QK^{T}}{\sqrt{d}}\right)\in R^{L\times L}$$

Each element $A_{i}\left(p,q\right)$ of the matrix represents the association between the token and the $q^{th}$ token in the $i^{th}$ pair of frames. The use of the softmax operation ensures that the sum of attention weights for each row equals 1.

In the video keyframe selection task, our focus lies in the overall frame-level difference rather than the detailed output at the token level. To this end, an aggregation operation can be applied to the attention matrix $A_{i}$ to derive a scalar representing the inter-frame similarity $S_{i}$. One of the most commonly used methods is to compute either the mean or the maximum value of the elements in $A_{i}$, as in Equation (3).

(3) $$S_{i}=\mathrm{Pool}\left(A_{i}\right)$$

Here, Pool represents a pooling operator, such as average or max pooling. A larger $S_{i}$ indicates a closer similarity between the two frames, whereas a smaller $S_{i}$ reflects a more substantial content difference between the frames. As highlighted in this investigation, to emphasize the identification of keyframes with “the greatest changes”, we define the difference function as depicted in Equation (4).

(4) $$diff_{i}=1-S_{i}$$

The difference function $d_{i}$ intuitively reflects the “magnitude of change” between frame $I_{i}$ and frame $I_{i+1}$. When $d_{i}$ takes a relatively large value (i.e., when similarity is very low), it indicates a significant visual transition at position *i*, often corresponding to a new scene or action turning point.

To select keyframes that best represent significant content changes, the difference sequence $\left\{d_{1},d_{2},\ldots ,d_{N-1}\right\}$ can be sorted, or the values can be compared to a threshold τ. Frames with the highest scores or those exceeding the threshold are selected for further consideration. When τ is set as a reference threshold for identifying peaks of change in a video, the final keyframe set *K* is represented as in Equation (5).

(5) $$K=\{I_{i+1}|diff_{i}>\tau \}$$

Here, *K* serves as the final set of selected keyframes, capturing the most significant scene changes.

We consider the relationships among all tokens within frames, rather than merely conducting comparisons at corresponding positions, which enables the capture of complex spatial variations. Through aggregation operations on the attention matrix $A_{i}$, the analysis can be elevated from token-level granular changes to frame-level holistic differences, thereby providing a more comprehensive understanding of temporal dynamics within the video sequence.

### 4.3. Multimodal Attention Fusion

**Figure 3:**
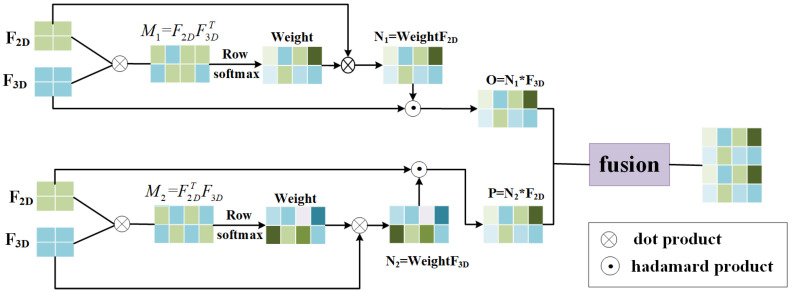
Multimodal attention fusion. The network architecture demonstrates our proposed dual-modality attention fusion mechanism designed to integrate 2D and 3D visual representational information. The architecture processes two input feature streams: F2D (2D CNN features extracted using ResNet152) and F3D (3D CNN features extracted using I3D network), both enhanced by the Self-Attention Embedding module. The fusion process employs attention weight computation through matrix operations, followed by row-wise softmax normalization to generate attention distributions. The mechanism utilizes both dot product and Hadamard product operations to capture different types of feature interactions between the two modalities. The final fused representation combines the contextual information from both 2D spatial features and 3D spatiotemporal features.

A video is composed of a series of scenes or segments organized sequentially over time, and semantic relationships exist between these scenes or segment sequences. Specifically, the current segment may depend on information conveyed by the previous segment, or it may be highly correlated with subsequent segments. This reflects the interdependence of video segments and their contextual relationships [31,32,33]. In real-world scenarios, different types of representational information exhibit strong complementarity. Leveraging multi-source information can effectively uncover latent signals, offering both consistency and complementarity from various perspectives. Consistency refers to shared, common information, while complementarity represents independent or mutually exclusive types of information [34,35]. Therefore, we extract its 2D CNN features ϕ(V) using the ResNet152 network and its 3D CNN features ψ(V) using the I3D network. Using the Self-Attention Embedding module, we capture the representational information of each individual modality, obtaining the single-modality feature representations $F^{2D}$ and $F^{3D}$ enhanced by Self-Attention. Since $F^{2D}$ and $F^{3D}$ are derived from the Self-Attention Embedding module, they inherently incorporate the contextual information of their respective modalities. To integrate these two types of visual representational information, we propose a dual-modality attention fusion mechanism. The structure of the proposed network is illustrated in Figure 3.

To achieve dual-modality fusion, we begin by calculating a pair of matching matrices $M_{1}$ and $M_{2}\in R^{u\times u}$ on the two single-modality representations to capture their interaction information. The matching matrices are computed as shown in Equation (6).

(6) $$\begin{array}{c} M_{1}=F_{2D}F_{3D}^{T}\\ M_{2}=F_{2D}^{T}F_{3D} \end{array}$$

Secondly, the attention distribution weights, denoted as Weight, are calculated for the matching matrices using the softmax function, as shown in Equation (7). Weight represents the correlation between the 2D CNN and 3D CNN features extracted from the video. A higher weight value indicates stronger interactions between different visual features, underscoring the significance of the fused information.

(7) Weight=softmax(M)

Subsequently, the attention distribution weights Weight are multiplied with the single-modality feature matrices to obtain the final attention representation matrices $N_{1}$ and $N_{2}$, as defined in Equation (8).

(8) $$\begin{array}{c} N_{1}=\mathrm{Weight}\cdot F_{2D}\\ N_{2}=\mathrm{Weight}\cdot F_{3D} \end{array}$$

Finally, a multiplicative gating mechanism is employed to perform element-wise multiplication between the attention representation and the other single-modality representation, resulting in the intermodality attention matrices *O* and *P*, as defined in Equation (9). The matrices *O* and *P* are then combined to form the dual-modality attention fusion feature $F_{\mathrm{Att}}$, as described in Equation (10).

(9) $$\begin{array}{c} O=N_{1}*F_{3D}\\ P=N_{2}*F_{2D} \end{array}$$

(10) $$F_{\mathrm{Att}}=\mathrm{Concat}\left(O,\;P\right)$$

### 4.4. Synergetic Attention State Mamba

#### 4.4.1. Preliminary

Recently developed SSM architectural models [36,37,38], including Structured State Space Sequence Models (S4) and Mamba, establish their core mechanisms upon the theoretical foundations of traditional continuous systems. The operational principle of such systems involves utilizing an intermediate latent state variable $h\left(t\right)\in R^{N}$ as a bridge to achieve effective transformation from one-dimensional input functions or sequences x(t)∈R to corresponding outputs y(t)∈R, as described in Equations (11) and (12). This aforementioned process can be mathematically represented as a linear Ordinary Differential Equation (ODE).

(11) $$h^{\prime }\left(t\right)=Ah\left(t\right)+Bx\left(t\right)$$

(12) y(t)=Ch(t)

where $A\in R^{N\times N}$ represents the state matrix, while $B\in R^{N\times 1}$ and $C\in R^{N\times 1}$ denote the projection parameters.

Given the inherent characteristics of deep neural networks in processing discrete sequence data, continuous-time systems must undergo appropriate numerical discretization processes. Based on the Zero-Order Hold approximation, matrix exponential functions are employed for precise discretization transformation, which is defined following Equations (13) and (14).

(13) $$\bar{A}=\exp\left(\Delta A\right)$$

(14) $$\bar{B}={\left(\Delta A\right)}^{-1}\left(\exp(\Delta A)-I\right)\cdot \Delta B$$

where the discretization time step Δ serves as a critical hyperparameter that directly influences the fidelity of system dynamics characteristics. The discretized state space equation is subsequently transformed into the following recurrence relation.

(15) $$\begin{array}{cc} h_{k} & =\bar{A}h_{k-1}+\bar{B}x_{k}\\ y_{k} & =Ch_{k} \end{array}$$

Notably, modern SSM architectures achieve equivalent transformation between recursive and convolution computation through ingenious mathematical transformations. Specifically, the system’s impulse response can be represented as a structured convolution kernel.

(16) $$K=\left(C\bar{B},C\bar{A}\bar{B},\ldots ,C{\bar{A}}^{L-1}\bar{B}\right)$$

#### 4.4.2. SASM Block

Recent related studies have validated the exceptional performance of Mamba architecture across multiple critical application domains. Yang et al. proposed the SMamba framework [39], which effectively addresses real-time challenges in event-driven object detection through the introduction of sparsification mechanisms, providing novel technical pathways for high-frequency visual processing tasks. In the neuroimaging domain, Kannan et al. designed the BrainMT hybrid architecture [40] that ingeniously integrates Mamba’s sequential modeling advantages with Transformer’s attention mechanisms, successfully achieving precise modeling of long-range spatiotemporal dependencies in functional magnetic resonance imaging data. Xiao et al. constructed a frequency domain-enhanced Mamba model [41] that demonstrates significant performance improvements in remote sensing image super-resolution reconstruction tasks, providing compelling evidence for the effectiveness of combining frequency domain prior knowledge with State Space Models. Cui et al. proposed the MGCM framework [42] that achieves breakthrough progress in cancer prognosis analysis through deep integration of multimodal graph convolution with Mamba architecture.

Although the aforementioned studies demonstrate that SSMs achieve efficient long-sequence modeling through their elegant mathematical formulation, their fixed linear transformation mechanism constrains the model’s adaptive processing capabilities for input features. In video content understanding scenarios, different visual feature channels carry heterogeneous semantic information. For instance, specific channels may focus on capturing object details, while others are more oriented toward scene layout or motion patterns. Traditional SSMs lack the perceptual capability for such feature heterogeneity, rendering them unable to achieve dynamic feature selection and weight allocation, thereby limiting their performance potential in complex visual tasks. Based on this observation, we propose the Synergetic Attention State Mamba (SASM) module, which achieves synergetic optimization of channel attention and spatial state modeling through a dual-branch parallel architecture. The pseudocode of the algorithm is presented in Algorithm 2.
**Algorithm 2** Synergetic Attention State Mamba Block**Input:**  1:Input tensor $X\in R^{B\times C\times N}$    // Batch size *B*, Channels *C*, Sequence length *N***Output:** Output tensor $Y\in R^{B\times C\times N}$**Steps:**  1:// Step 1: Input Normalization  2:$X_{\mathrm{norm}}\leftarrow \mathrm{LayerNorm}\left(X\right)$  3:// Step 2: Attention Branch  4:$\mathrm{Att}\leftarrow \mathrm{Attention}(X_{\mathrm{norm}})$    // Multi-head attention or similar  5:FC_out←FC1(Att)    // Fully connected layer 1  6:GELU_out←GELU(FC1_out)    // GELU activation  7:FC2_out←FC2(GELU_out)    // Fully connected layer 2  8:$F_{\mathrm{att}}\leftarrow \mathrm{AddNorm}\left(\mathrm{FC2\_out}+\mathrm{Att}\right)$    // Residual connection with normalization  9:// Step 3: State Branch (SSM-based)10:$\mathrm{SSM\_out}\leftarrow \mathrm{SSM}(X_{\mathrm{norm}})$    // State Space Model (e.g., Mamba SSM)11:LN_out←LayerNorm(SSM_out)12:$F_{\mathrm{ssm}}\leftarrow \mathrm{FC}\left(\mathrm{LN\_out}\right)$    // Fully connected layer13:// Step 4: Synergetic Fusion14:$\mathrm{Prod}\leftarrow \mathrm{ElementWiseProduct}(F_{\mathrm{att}},F_{\mathrm{ssm}})$    // Channel-wise multiplication for synergy15:// Step 5: Output with Residual16:Y←Prod+X    // Add input residual for stability17:**Return** *Y*

**Figure 4:**
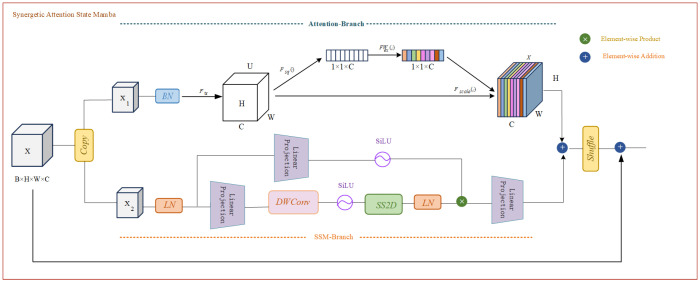
Synergetic Attention State Mamba block. This demonstrates the proposed Synergetic Attention State Mamba (SASM) module, which adopts a dual-branch parallel architecture to achieve synergetic optimization of channel attention and spatial state modeling. Unlike traditional SSMs with fixed linear transformation mechanisms, the SASM module integrates dynamic feature selection and weight allocation capabilities to handle heterogeneous semantic information across different visual feature channels.

As illustrated in Figure 4, the SASM module receives input features $X\in R^{B\times H\times W\times C}$ and simultaneously feeds them into the Attention-Branch and SSM-Branch for parallel processing. The Attention-Branch focuses on channel-level feature importance modeling, initially compressing the spatial dimensions through a global average pooling operation $F_{\mathrm{sq}}\left(\cdot \right)$, which aggregates the spatial information of each channel into a single global descriptor.

(17) $$z=F_{\mathrm{sq}}\left(X\right)=\frac{1}{H\times W}\sum_{i=1}^{H}\sum_{j=1}^{W}X_{i,j}\in R^{B\times 1\times 1\times C}$$

This global pooling strategy can effectively capture the statistical characteristics of each channel across the entire spatial domain, providing compact yet information-rich feature representations for subsequent channel importance assessment. Subsequently, a bottleneck structure is constructed through two-layer linear projections, incorporating SiLU activation functions to learn complex nonlinear dependencies among channels.

(18) $$A=F_{\mathrm{scale}}\left(F_{\mathrm{sq}}\left(X\right)\right)=\sigma \left(W_{2}\cdot \mathrm{SiLU}\left(W_{1}\cdot z\right)\right)$$

where $W_{1}\in R^{C/r\times C}$, $W_{2}\in R^{C\times C/r}$, and *r* represents the compression ratio. The first linear projection reduces parameter count and enhances generalization capability through dimensionality reduction operations (setting r=16), while the SiLU activation function exhibits superior gradient flow characteristics and nonlinear expression capabilities compared to conventional ReLU. The second projection layer restores the feature dimensionality to the original channel count, ultimately constraining the output values within the [0,1] range through the Sigmoid activation function σ(·), thereby generating normalized channel attention weights. This approach not only effectively controls model complexity but also facilitates the network’s learning of more robust and semantically rich inter-channel relational representations by introducing a moderate information bottleneck, thus achieving precise quantification and dynamic adjustment of feature importance across different channels.

The SSM-Branch focuses on efficient modeling of spatial long-range dependencies. Input features first undergo layer normalization (LN) for feature standardization, an operation that stabilizes the training process and alleviates gradient vanishing problems, ensuring numerical stability for subsequent state space modeling. The normalized features are then subjected to linear projection for feature transformation, mapping the input features to a representation space suitable for state space processing.

(19) $$X_{\mathrm{proj}}=\mathrm{Linear}\left(\mathrm{LN}(X)\right)$$

Subsequently, the local spatial receptive field is enhanced through depthwise separable convolution (DWConv):

(20) $$X_{\mathrm{local}}=\mathrm{DWConv}\left(X_{\mathrm{proj}}\right)$$

Depthwise separable convolution exhibits lower computational complexity compared to standard convolution while effectively capturing local spatial patterns, providing rich local contextual information for subsequent global state space modeling. This local–global hierarchical modeling strategy ensures that the model can perceive fine-grained spatial details while establishing long-range spatial dependencies. Subsequently, the transformed features are fed into the two-dimensional state space module (SS2D) for long-range spatial dependency modeling:

(21) $$X_{\mathrm{spatial}}=\mathrm{SS}2D\left(X_{\mathrm{local}}\right)$$

**Figure 5:**
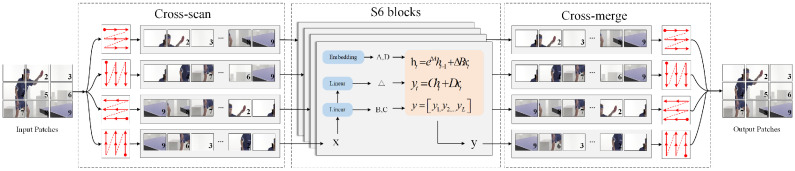
The internal modeling process of SS2D. This demonstrates the internal modeling process of the SS2D (two-dimensional state space) module, which extends traditional one-dimensional State Space Models to two-dimensional space to effectively capture long-range dependencies across spatial domains. The architecture demonstrates the cross-scan mechanism that processes input video frames in multiple directions, followed by S6 blocks that maintain recursive updates of hidden states in both horizontal and vertical directions. The process includes cross-merge operations that integrate features from different scanning directions. Unlike self-attention mechanisms with quadratic complexity O((HW)^2^), the SS2D design achieves linear time complexity O(HW), significantly reducing computational overhead and making it particularly suitable for rapid processing of large batches of video frames.

SS2D captures inter-frame temporal dependencies while inheriting the linear complexity of S6, which is crucial for generating accurate and coherent video captions. SS2D comprises three components: the Temporal Scanning and Merging operation (TSM), the visual S6 block, and the feature fusion operation. Figure 5 intuitively illustrates the internal mechanism of SS2D in video caption generation. Specifically, the temporal scanning and merging operation initially unfolds the video frame sequence into visual feature streams along four distinct directions (forward temporal, backward temporal, intra-scene keyframes, and scene transition points). Subsequently, the visual S6 block processes all sequences to extract spatiotemporal features, ensuring comprehensive capture of dynamic changes and scene information within the video content. Finally, the feature fusion operation integrates the output features from the four directions, constructing rich visual-semantic representations to generate accurate caption descriptions that are highly aligned with the video’s visual content.

The SS2D module (Figure 5) represents an extension of traditional one-dimensional State Space Models to two-dimensional space, which effectively captures long-range dependencies across the entire spatial domain with linear time complexity O(HW) by maintaining recursive updates of hidden states in both horizontal and vertical directions. Compared to the quadratic complexity $O({\left(\mathrm{HW}\right)}^{2})$ of self-attention mechanisms, this design significantly reduces computational overhead, making it particularly suitable for processing high-resolution video frames. The collaborative fusion mechanism achieves feature integration of the two branches through element-wise multiplication operations.

(22) $$X_{\mathrm{proj}}=\mathrm{Linear}\left(\mathrm{LN}(X)\right)$$

where ⊙ denotes the element-wise multiplication operation. This fusion strategy enables attention weights *A* to perform dynamic weighting of spatial features from the SSM-Branch along the channel dimension, achieving channel-sensitive spatial feature selection. Specifically, spatial features of important channels are amplified while contributions from redundant channels are suppressed, thereby enhancing the discriminative capability and semantic consistency of the overall feature representation. This gating mechanism effectively combines the selectivity of attention with the temporal modeling capability of State Space Models, achieving complementary advantages.

### 4.5. Adaptive Attention Module

The Adaptive Attention Module serves as the core component connecting multimodal video features with text generation in our video captioning framework. During the caption generation process, this module dynamically computes attention weights between the LSTM decoder’s hidden states and the fused multimodal features $F^{fused}$, enabling context-aware text generation that adaptively focuses on relevant visual content at each decoding step. During the network decoding stage, an adaptive attention mechanism [43] is incorporated to extract attention-enhanced weights $e_{t}$ by combining the attention-fused visual features *F* with the hidden state outputs $h_{t}$ of the parallel LSTM decoder [44] at each time step. This mechanism adaptively adjusts the correlation ratio between the multimodal semantic hidden information $g_{t}$ and the adaptive attention outputs $e_{t}$, focusing more effectively on the key contents of the multimodal representations encoded by the mul-feature. The adaptive attention computation process is as follows:

First, the enhanced information for the adaptive attention mechanism is calculated using Equations (23)–(25), providing attention information under the fusion mode for the adaptive attention module.

(23) $$z_{t}=W_{z}^{T}\tanh\left(W_{v}F+\left(W_{h}h_{t}\right)1^{T}\right)$$

(24) $$\alpha_{t}=\mathrm{softmax}\left(z_{t}\right)$$

(25) $$e_{t}=\sum^{k}\alpha_{ti}f_{ti}^{\prime }$$

At this point, $h_{t}\in R^{m}$, $W_{z}\in R^{k}$, $W_{v}\in R^{k\times m}$, and $W_{h}\in R^{k\times m}$ represent the weight parameters, and *F* denotes the output feature vector from the modality attention fusion. To ensure dimensional alignment during matrix addition in Equation (23), $1^{T}$ is employed for dimension adjustment; $1^{T}\in R^{k}$, where $\alpha_{t_{i}}$ is a vector with all elements equal to 1; $\alpha_{t}\in R^{k}$; and $f_{t_{i}}^{\prime }$ represents the $i^{th}$ feature of the dual-modality fused attention at time step *t*.

Secondly, the visual sentinel module $\lambda_{t}$ in Equation (26) adaptively regulates the output $r_{t}$ of the attention mechanism. During the adaptive regulation process, the determination of $r_{t}$ depends on whether it is more influenced by the information $e_{t}$, reinforced through adaptive attention, or by the hidden information $g_{t}$ captured based on dual-modality fused attention.

(26) $$r_{t}=\lambda_{t}g_{t}+\left(1-\lambda_{t}\right)e_{t}$$

The computation of the visual sentinel $\lambda_{t}$ is defined in Equation (27).

(27) $$\lambda_{t}=\mathrm{softmax}\left(\{z_{i};{\hat{z}}_{i}\}\right)\left[k+1\right]$$

Here, $\lambda_{t}$ is obtained from the last element of the output result of the softmax function, while ${\hat{z}}_{i}$ is derived from the fusion of the multimodal fused hidden information $h_{t}$ and the visual fused hidden information $g_{t}$.

Unlike static attention that treats all decoding steps uniformly, our adaptive attention considers the semantic evolution of the generated text sequence $w_{1},w_{2},\ldots ,w_{t-1}$ to determine which visual aspects are most relevant for predicting the next word $w_{t}$. This is achieved by conditioning the attention computation on the decoder’s hidden state $h_{t}$, which encodes the contextual information from previously generated words.

## 5. Experimental Analysis

### 5.1. Experimental Platform

The hardware configuration for our study involves training the model initially on a cloud server equipped with an Intel(R) Xeon(R) Gold 5118 CPU @ 2.30 GHz and an NVIDIA Tesla V100 GPU, followed by deployment of the final model to an edge camera utilizing the NVIDIA Jetson TX2 for evaluating runtime speed and performance. It has a dual-core Denver 2 64-bit CPU and a quad-core ARM A57 Complex graphics processor architecture with 256 CUDA cores for parallel computing and a Pascal graphics acceleration architecture. In addition, the CPUs and the graphics processor share 8 GB of RAM. For model assessment, we employed the MSR-VTT and MSVD datasets, each comprising videos paired with their corresponding human-annotated descriptive sentences (ground truth). In terms of evaluation metrics, this work adopts the Bilingual Evaluation Understudy (BLEU) [45], the recall-oriented understudy for the evaluation of the girth with the longest common sequence (ROUGE-L) [46], the metric for the evaluation of translation with explicit ordering (METEOR) [47], and the Consensus-based Image Description Evaluation (CIDEr) [48]. The training platform and the experimental runtime environment for the model are given in Table 1.

### 5.2. Data Preprocessing

The proposed model utilizes the MSVD [49] and MSR-VTT [26] datasets. First, the extracted video frames are formatted and resized to 224 × 224 pixels. Then, 2D CNN features for each frame are extracted using the ResNet model, which incorporates channel attention. Additionally, 3D CNN features for the videos are extracted using the I3D pre-trained model, integrating optical flow and 3D spatial information. Finally, audio features corresponding to each video are extracted. The audio features are obtained using VGGish, a large-scale pre-trained model proposed by Google’s Speech Understanding team. VGGish is trained on the extensive AudioSet dataset [10], offering strong generalization capabilities. For the textual descriptions in the datasets, the following steps are performed: First, sentences are tokenized using the Stanford NLP Toolkit, then truncated to 20 words, with punctuation removed and all words converted to lowercase. Next, the descriptions are tokenized, and all vocabularies are aggregated. A word frequency threshold is set to five, with words exceeding the threshold included in the vocabulary, while low-frequency words are excluded. Special tokens such as “<bos>“, “<eos>“, “<pad>“, and “<nan>“ are added to the vocabulary. Finally, the generated vocabulary is numerically indexed, and each textual description is vectorized into a format suitable for computation.

### 5.3. Experimental Parameter Settings

In the Self-Attention Embedding layer, the pooling kernel size is set to 3 × 3, and the output dimensions of the adaptive attention mechanism and other network components are all set to 1024.

During the model training, the Adam optimizer is utilized, and the batch size is set to 64. The initial learning rate is set to 0.0001, and it decays by a factor of 0.1 if the evaluation score on the validation set does not improve for 20 epochs after every 100 iterations. Gradient clipping is applied during backpropagation to prevent gradient explosion during training. During the description generation phase, the Dropout technique is employed, with a dropout rate of 0.5 to prune neurons in the network model. This approach reduces the interdependence among neurons in the video content description model, improving the generalization capability of the model while preventing overfitting.

### 5.4. Analysis of Experimental Results

**Figure 6:**
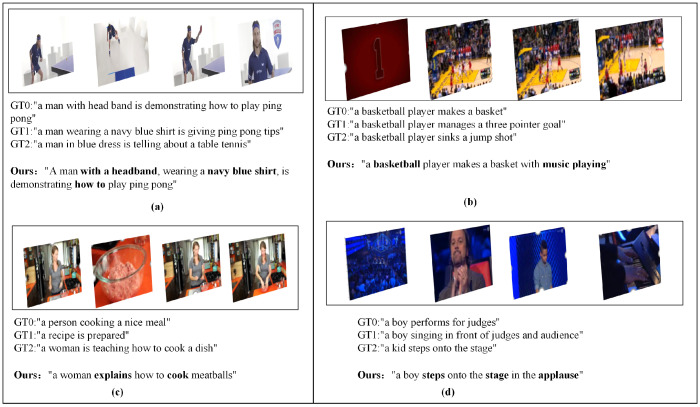
Test examples of the model. This demonstrates representative test examples of the model across different video content scenarios. Each example (**a**–**d**) displays sequential video frames, accompanied by multiple ground truth (GT) descriptions and the descriptions generated by the model for the videos. The comparison between ground truth annotations and model outputs demonstrates the system’s capability to generate contextually appropriate and semantically accurate video descriptions across different domains and activity types.

To validate the effectiveness of the proposed video content description generation model, a comparative analysis was conducted against state-of-the-art models, including OA-BTG, POS + VCT, ORG-TRL (IRV2 + C3D), and SGN. The evaluation metrics used for comparison include BLEU, ROUGE, METEOR, and CIDEr. The detailed qualitative results are presented in Table 2 and Table 3, while the quantitative results are illustrated in Figure 6.

From the perspective of the short video description generation task, although the MSVD dataset features shorter video durations and relatively simpler scenes, it still places high demands on the model’s ability to generate accurate descriptions and capture core actions or scene elements. As shown in Table 2, the proposed model (“ours”) achieves the highest or near-highest scores across BLEU-4, METEOR, ROUGE-L, and CIDEr metrics, surpassing advanced methods such as ViT/L14 and ORG-TRL. Notably, “ours” demonstrates a significant advantage in metrics like METEOR and CIDEr, which emphasize semantic and content matching more. This indicates the model’s superior ability to comprehend video actions and semantic information, enabling precise descriptions of key content. Further comparisons reveal that compared to alternative methods requiring complex features or external knowledge augmentation; “ours” exhibits robust performance improvements under identical or similar feature conditions, suggesting that the multimodal feature fusion and temporal-context modeling strategies employed in “ours” effectively enhance the capture of core actions, events, and semantic information within short video scenarios.

The MSR-VTT dataset encompasses more diverse scenes and multimodal information, with longer video durations and more complex content, imposing greater demands on a model’s ability to integrate temporal modeling, action recognition, scene understanding, and language generation. As shown in Table 3, the proposed “ours” model also achieves top-tier or leading results across BLEU-4, METEOR, ROUGE-L, and CIDEr metrics. Particularly for BLEU-4 and CIDEr, where performance differences indicate a model’s ability to capture critical semantic words and video attributes, “ours” outperforms other prominent methods, demonstrating its superiority in temporal dependency modeling and visual–semantic alignment. Compared to methods also reliant on visual features or knowledge graphs (e.g., ViT/L14, TextKG and STLF), the proposed model achieves more effective cross-modal information flow by improving multimodal feature fusion, temporal clue extraction, and language decoding strategies. It balances the diversity of video content with the accuracy of language expression during the generation process.

To validate the applicability and effectiveness of our proposed model in practical IoT edge computing environments, we deployed the trained model on edge devices for inference testing to evaluate its actual performance under resource-constrained conditions. Based on the performance comparison presented in Table 4, our proposed model demonstrates superior efficiency on the edge device platform (NVIDIA Jetson TX2) compared to existing state-of-the-art approaches. Specifically, our model achieves the lowest memory footprint of 2.97 GB, representing a reduction of 7.2% and 4.2% compared to Gated-ViGAT (3.2 GB) and SLSMedvc (3.1 GB), respectively. More importantly, our approach exhibits the fastest inference time of 0.3000 seconds, outperforming both Gated-ViGAT (0.3483 s) and SLSMedvc (0.3090 s) by 13.9% and 2.9%, respectively.

These improvements are particularly significant for edge deployment scenarios where computational resources are constrained and real-time processing is critical. Compared to Vision Transformer-based works, our Mamba-based architecture demonstrates superior computational efficiency and resource utilization on edge devices. While ViT architectures face significant computational and memory bottlenecks when processing long sequences due to their quadratic complexity in self-attention mechanisms, our Mamba-based approach effectively mitigates this issue through linear complexity state space modeling. The synergistic optimization achieved through our Synergetic Attention State Mamba Block not only maintains strong representational capabilities but also significantly reduces computational overhead, making it exceptionally well-suited for practical edge computing applications in video understanding tasks.

To comprehensively evaluate the performance of the proposed model, we implemented multiple network structure versions within the same experimental environment. These versions integrated different functional modules, including support for multimodal data, uniform frame sampling strategies, cross-attention frame selection mechanisms, miniGPT-based visual localization, and adaptive attention mechanisms. By conducting ablation experiments on the same dataset, we could independently examine each module’s contribution to the overall performance and its impact on the quality of video descriptions. As depicted in Table 5 and Table 6, experimental results demonstrate the performance of each module across various evaluation metrics, providing strong evidence for informed decisions in subsequent model selection and optimization.

**Component Contribution Analysis:** The ablation study results demonstrate that the contribution of each component to model performance exhibits a distinct progressive pattern. The miniGPT method based on large-scale pre-trained language models outperforms traditional cross-attention mechanisms on both benchmark datasets (MSVD: BLEU-4 59.2 vs. 58.1; MSR-VTT: BLEU-4 40.3 vs. 39.2), fully demonstrating the inherent advantages of large-scale pre-trained language models. The combination of the visual localization frame selection strategy with the miniGPT framework produces significant synergistic effects (MSR-VTT: BLEU-4 improvement from 40.3 to 42.3), while the introduction of adaptive attention mechanisms further enhances model performance, particularly when combined with the miniGPT architecture (9.4% BLEU-4 improvement on MSR-VTT). Although the multi-component collaborative configuration already exhibits superior performance, the final integration of the proposed SASM module achieves optimal performance across all evaluation metrics, reaching BLEU-4 61.6 and CIDEr 123.9 on the MSVD dataset, and BLEU-4 45.1 and ROUGE-L 63.8 on MSR-VTT. That is, these results confirm the existence of significant positive synergistic effects among the proposed components.

**Cross-Dataset Performance Analysis:** The experimental results on two benchmark datasets reveal the adaptive characteristics of the proposed model in processing video content of different complexities. The model’s overall performance on the MSVD dataset significantly exceeds that on MSR-VTT (baseline configuration BLEU-4: 58.1 vs. 39.2), reflecting the inherent differences in video–text alignment difficulty between the two datasets. Notably, advanced components demonstrate a greater relative contribution on MSR-VTT, the more challenging dataset. For instance, the combination of adaptive attention mechanism with miniGPT achieves a 9.4% BLEU-4 improvement on MSR-VTT compared to only 2.2% on MSVD, indicating that complex datasets better highlight the technical value of advanced components. Notably, the proposed SASM component under simplified configuration shows remarkable performance enhancement on MSR-VTT (3.7% BLEU-4 improvement compared to baseline miniGPT), further validating its superior capability in handling complex video content. The experimental results also demonstrate that SASM consistently provides stable performance gains across different configuration combinations, improving CIDEr by 1.9% on MSVD and ROUGE-L by 1.3% on MSR-VTT. This finding fully demonstrates the critical value of the proposed SASM module as the final refinement component of the model architecture, providing comprehensive and significant performance improvements for video description tasks.

## 6. Concluding Remarks

In this work, we present EdgeVidCap, a lightweight video captioning model tailored explicitly for resource-constrained IoT edge cameras. Our approach addresses the critical challenge of deploying sophisticated video understanding algorithms on edge devices while maintaining competitive performance. The key innovation lies in the Synergetic Attention State Mamba (SASM) module, which synergistically combines channel attention mechanisms with State Space Models to achieve efficient spatiotemporal feature modeling. Additionally, our adaptive attention-guided LSTM decoder dynamically adjusts feature weights to generate semantically rich and contextually accurate textual descriptions. Comprehensive experiments on MSR-VTT and MSVD datasets demonstrate that EdgeVidCap achieves competitive performance compared to state-of-the-art methods while significantly reducing computational complexity and parameter count, making it well-suited for real-time deployment on IoT edge devices. Looking ahead, extending our framework to multi-lingual caption generation and incorporating domain-specific knowledge for specialized applications such as surveillance or autonomous driving represents another valuable direction. Finally, developing more sophisticated attention mechanisms that can better capture fine-grained temporal dynamics and object interactions in complex video scenarios remains an important area for future exploration. These developments will continue to advance the practical deployment of intelligent video understanding systems in edge computing environments.

Nevertheless, several limitations warrant consideration. The lightweight architecture may struggle with highly complex scenes involving numerous objects or rapid transitions, and the model’s ability to maintain contextual coherence across extremely long video sequences remains constrained by the inherent limitations of State Space Models for extended temporal modeling. Additionally, certain content types such as abstract videos or highly technical domains may challenge the current framework’s representational capacity. Looking ahead, extending our framework to multi-lingual caption generation and incorporating domain-specific knowledge for specialized applications such as surveillance or autonomous driving represents another valuable direction. Finally, developing more sophisticated attention mechanisms that can better capture fine-grained temporal dynamics and object interactions in complex video scenarios remains an important area for future exploration. These developments will continue to advance the practical deployment of intelligent video understanding systems in edge computing environments.

## Figures and Tables

**Table 1:** Experimental platform and environment configuration.

Experimental Platform	Environment Configuration
RAM	128 GB
CPU	Intel(R) Xeon(R) Gold 5118 CPU @ 2.30 GHz (Intel Corporation, Santa Clara, CA, USA)
GPU_Memory	32 GB
GPU	NVIDIA Tesla V100 (NVIDIA Corporation, Santa Clara, CA, USA)
Driver	NVIDIA CUDA 11.0
Deep Learning Acceleration Library	cuDNN-V8.0.5
Deep Learning Framework	PyTorch 1.6
Inference device	NVIDIA Jetson TX2 (NVIDIA Corporation, Santa Clara, CA, USA)

**Table 2:** Comparative experiments of models on the MSVD dataset.

Model	BLEU-4	METEOR	ROUGEL	CIDEr
OA-BTG [21]	45.8	33.3	-	73.0
POS+VCT [50]	52.8	36.1	71.8	87.8
ORG-TRL (IRV2 + C3D) [51]	54.3	36.4	73.9	95.2
SGN [52]	52.8	35.5	72.9	94.3
TextKG [53]	60.8	38.5	75.1	105.2
MAN [54]	60.1	37.1	74.6	101.9
VIT/L14 [55]	60.1	41.4	78.2	121.5
GSEN [56]	58.8	37.6	75.2	102.5
STLF [57]	60.9	40.8	77.5	110.9
ours	**61.6**	**42.7**	**96.1**	**123.9**

**Table 3:** Comparative experiments of models on the MSR-VTT dataset.

Model	BLEU-4	METEOR	fROUGEL	CIDEr
OA-BTG [21]	41.4	28.2	-	46.9
POS+VCT [50]	42.3	29.7	62.0	49.1
ORG-TRL (IRV2 + C3D) [51]	43.6	28.8	62.1	50.9
SGN [52]	40.8	28.3	60.8	49.5
TextKG [53]	43.7	29.6	62.4	52.4
MAN [54]	42.5	28.6	62.2	50.4
VIT/L14 [55]	44.4	30.3	63.4	57.2
GSEN [56]	42.9	28.4	61.7	51.0
STLF [57]	**47.5**	30.3	63.8	54.1
ours	45.1	**30.6**	**63.8**	**58.6**

**Table 4:** Performance comparison of models on edge device NVIDIA Jetson TX2.

Model	Memory Usage	Inference Time(s)
Gated-ViGAT	3.2 GB	0.3483
SLSMedvc	3.1 GB	0.3090
ours	**2.97 GB**	**0.3000**

**Table 5:** Ablation study of the model on the MSVD dataset.

Multimodal Features	Uniform Frame Sampling	Cross-Attention Frame Selection	miniGPT Visual Localization	Adaptive Attention	SASM	BLEU-4	METEOR	ROUGEL	CIDEr
✔	✔					58.1	40.3	93.7	115.4
✔		✔				59.2	41.0	94.1	118.3
✔	✔		✔			58.6	41.4	93.8	118.6
✔		✔	✔			60.2	41.7	95.2	120.8
✔	✔			✔		59.9	41.2	95.1	120.5
✔		✔		✔		60.5	41.8	95.4	121.3
✔	✔		✔	✔		61.1	42.2	95.7	121.6
✔			✔		✔	60.0	41.6	94.8	120.2
✔	✔	✔	✔		✔	60.9	42.1	95.5	122.4
✔	✔	✔	✔	✔	✔	**61.6**	**42.7**	**96.1**	**123.9**

**Table 6:** Ablation study of the nodel on the MSR-VTT dataset.

Multimodal Features	Uniform Frame Sampling	Cross-Attention Frame Selection	miniGPT Visual Localization	Adaptive Attention	SASM	BLEU-4	METEOR	ROUGEL	CIDEr
✔	✔					39.2	27.8	60.1	52.7
✔		✔				40.3	28.1	60.7	53.5
✔	✔		✔			39.5	27.9	60.0	54.3
✔		✔	✔			42.3	28.7	61.2	55.1
✔	✔			✔		42.0	29.1	60.4	55.8
✔		✔		✔		44.1	29.8	63.1	57.4
✔	✔		✔	✔		44.6	30.1	63.0	58.2
✔			✔		✔	41.8	29.0	61.8	55.7
✔	✔	✔	✔		✔	43.9	29.8	62.9	57.3
✔	✔	✔	✔	✔	✔	**45.1**	**30.6**	**63.8**	**58.6**

## Data Availability

The raw data supporting the conclusions of this article will be made available by the authors on request.
